# A Novel CTLA-4 affinity peptide for cancer immunotherapy by increasing the integrin αvβ3 targeting

**DOI:** 10.1007/s12672-022-00562-6

**Published:** 2022-10-04

**Authors:** Ying Zhou, Shuyi Song, Baomei Yuan, Yahong Wu, Yanfeng Gao, Guangming Wan, Guodong Li

**Affiliations:** 1 School of Life Sciences, Zhengzhou University, Zhengzhou, 450001 China; 2 School of Pharmaceutical Sciences (Shenzhen), Sun Yat-Sen University, Guangzhou, 510006 China; 3 Henan Key Laboratory of Bioactive Macromolecules, Zhengzhou University, Zhengzhou, 450001 China; 4 International Joint Laboratory for Protein and Peptide Drugs of Henan Province, Zheng Zhou University, Zhengzhou, 450001 China; 5 The First Affiliated Hospital of Zhengzhou University, Zhengzhou, 450001 China

**Keywords:** CTLA-4, Immune checkpoint inhibitors, Peripheral immunity, Peptide, Side effects

## Abstract

Immune checkpoint inhibitors (ICIs) are changing all aspects of malignant tumour therapy as an immunotherapy subverter in oncology. However, the current ICIs might induce systemic immune activation in other tissues and organs since they are not tumour-specific, causing the immune system to attack some normal tissues and organs of the human body. The toxicity can also amplify greatly although combined immunotherapy for cancer has increased the curative efficacy. The LC4 peptide was modified to improve its tumour-targeting ability and reduce peripheral immune system activation, which was obtained through phage display peptide library screening and could block the CTLA-4/CD80 interaction. The LC4 peptide as a result, like other ICIs, exerts anti-tumour effects by refreshing T cell function, and also activates the peripheral immune system. We used the PLGLAG peptide as a linker at the C-terminal of LC4 to connect with a tumour-targeting peptide RGD to increase the tumour tissue targeting ability, and obtain LC4-PLG-RGD. Further experiments demonstrated that the anti-tumour LC4-PLG-RGD activity was better than LC4 in vivo, and the ability to activate the peripheral immune system was weakened. In conclusion, LC4-PLG-RGD can increase the ICIs tumour-targeting and reduce excessive peripheral tissue immune activation, thereby reducing the side effects of ICIs, while increasing their anti-tumour efficacy. This study confirmed that enhanced ICI tumour targeting can effectively reduce immune-related adverse reaction occurrence.

**Supplementary Information:**

The online version contains supplementary material available at 10.1007/s12672-022-00562-6.

## Introduction

Immune checkpoint inhibitors (ICIs) have become a central cancer treatment pillar, with almost half of all patients with metastatic cancer in economically developed countries eligible for ICIs treatment [1]. ICIs are also frequently used in combination regimens as more and more become available, which greatly improves the ICI treatment response rate. However, the immune-related adverse events (irAE) associated with ICI combination were also increased significantly, e.g. treatment with a combination of occurrence of ipilimumab and nivolumab increased severe side effects by 2–fourfold compared with monotherapies alone [2]. The immune activation of most immune-related adverse reactions (irAE) are related to the activity required for an anti-tumour immune response. Therefore, how to enhance the anti-tumour ICI activity while reducing the irAE occurrence is an urgent drug development problem.

Immune checkpoint therapy can inhibit immune checkpoint activity, release immune brakes in the tumour microenvironment, and reactivate T cell immune tumour response, thus achieving anti-tumour effect. Therefore, increasing ICI enrichment in the tumour microenvironment and decreasing peripheral immune system activation is an effective method to realise this idea. It was reported that dual-targeted antibodies improve T lymphocyte infiltration in tumour tissues and thus achieve a stronger anti-tumour effect than single targeted antibodies [3], while there are few reports on reducing peripheral immune system activation.

CTLA-4 plays a negative regulatory role in the initial T cell activation stage, and is mainly expressed on activated CD8^+^ T cells and CD4^+^T cells, and constitutively expressed on Treg cells [4, 5]. Blocking CTLA-4 can reverse and restore depleted T cell function, improve proliferation and T cell effector capacity, and inhibit tumour growth significantly [6]. The CTLA-4 monoclonal antibody ipilimumab was officially approved for unresectable stage III / IV metastatic melanoma treatment by the FDA. IrAEs from anti-CTLA-4 agents are dose-dependent and occur more frequently, which limit their clinical application.

Integrin is an important cell adhesion receptor, which is highly expressed in tumour vascular endothelial cells and some tumour cells [7]. RGD peptide has dual targeting, which can simultaneously target tumour cells and tumour endothelial cells by specific affinity with integrin αvβ3. It has been used to deliver anti-tumour drugs or contrast agents in tumour therapy and diagnosis [8, 9]. Matrix Metalloproteinase-2 (MMP-2) is a protease involved in ECM degradation in tumours. PLGLAG is its restriction site. MMP-2 is highly expressed in almost all tumour tissues. It is sensitive and highly specific in tumour tissues, which has a good application prospect in cancer treatment [10, 11]. Hong Xia Wang et al. confirmed that PLGLAG can be cut off when it enters tumour tissue [12]. Therefore, PLGLAG is the restriction site of MMP-2, and is a widely used linker, which can release the modified drug in tumour tissue.

We first obtained CTLA-4 affinity peptide LC4 by phage display peptide library screening in this study. LC4 peptide has been demonstrated to effectively block CTLA-4 /B7 protein interactions and has good anti-tumour effect in vitro and in vivo. PLGLAG was used to connect LC4 with RGD, a tumour-targeting peptide sequence with high affinity for integrin αvβ3, and obtain a modified peptide LC4-PLG-RGD. LC4-PLG-RGD as a result has better anti-tumour effect, could reduce peripheral immune system activation, and reduce irAEs produced in CTLA-4 treatment.

## Results

### Acquisition of CTLA-4 affinity peptide and determination of its affinity with CTLA-4 molecule

#### Screening and synthesis of CTLA-4 affinity peptides

Phage display peptide library technology was used to screen affinity peptides that bind to CTLA-4. Bio-panning five rounds on the hCTLA-4 protein were performed to obtain the hCTLA-4 binding peptides. Only eight peptides were identified and the amino acid sequences with the enriched phage clones were shown in Table 1 after sequencing of the randomly selected 84 phage clones. The eight peptides (LC1-LC8) were synthesised and validated (data not shown).

**Table 1:** Clone frequency and selected phage peptide sequences

Name	Peptide Sequence	Frequency(n/84)
LC1	RWKDTAYALTNN	4
LC2	HHLRIPYALDQT	2
LC3	SWHWHTHVRHQM	3
LC4	WGHSHFSHWKGR	3
LC5	SHRWQVWSRDRA	2
LC6	ASANDNRLRYTY	2
LC7	LDRPSSLAHLAS	2
LC8	RHHSSNPRDTAP	2

#### Affinity measurement between hCTLA-4 binding peptides and hCTLA-4 protein

**Fig. 1:**
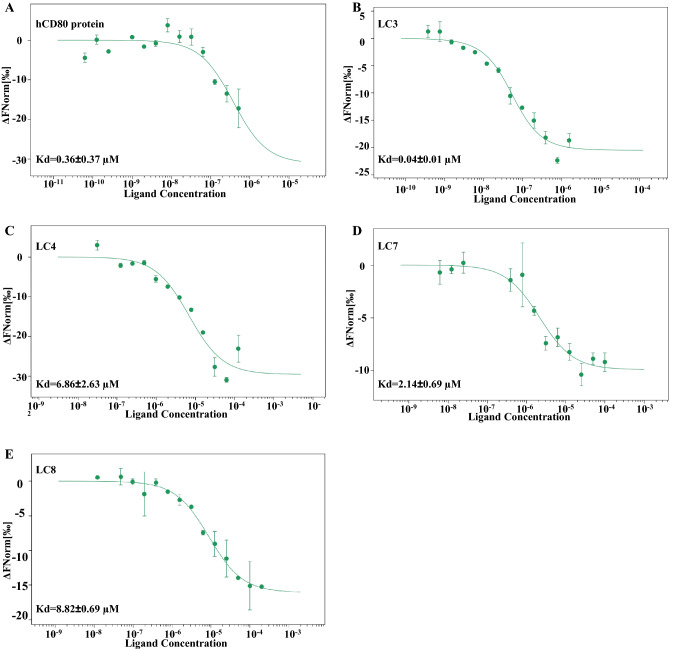
Binding peptide to hCTLA-4 protein detection by MST. **A**–**E** The MST binding curves of human CTLA4 binding peptides to hCTLA4

The microscale thermophoresis (MST) binding curves of hCTLA-4 binding peptides to hCTLA-4 were shown in Fig. 1. The binding of hCTLA-4 binding peptides to hCTLA-4 for the Kd values were shown in Table 2. The results showed that LC3, LC4, LC7, LC8 could bind hCTLA-4. These four peptides were selected for follow-up experiments to verify their specific affinity.

**Table 2:** The human CTLA4 binding peptides to hCTLA-4 protein affinity was measured by MST

Name	Kd(μM)
hCD80	0.36 ± 0.37
LC1	NB
LC2	NB
LC3	0.04 ± 0.01
LC4	6.86 ± 2.63
LC5	NB
LC6	NB
LC7	2.14 ± 0.69
LC8	8.82 ± 0.69

(*NB = No Binding)

## The affinity peptide interferes with the CTLA-4 to its ligands CD80 and CD86 binding by specifically binding to CTLA-4

### Affinity peptides specifically bind CTLA-4 molecules on cells, impairing the binding of CTLA-4 to its ligand

**Fig. 2:**
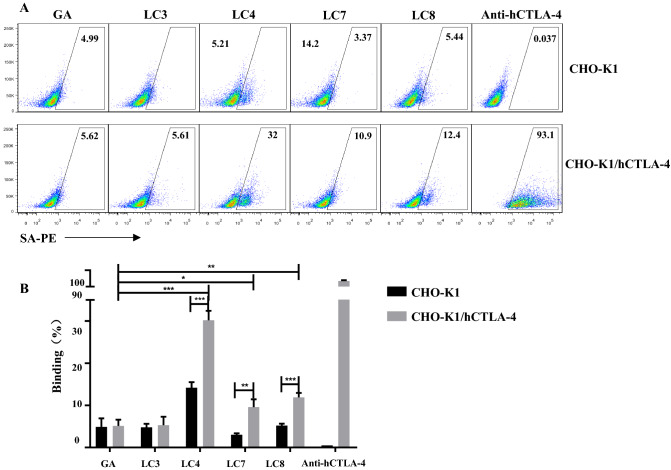
The specific binding of CTLA-4 binding peptides to CTLA4 was determined via cell-based binding assay. Biotin-labelled CTLA-4 binding peptides at 200 μM were incubated with CHO-K1/ hCTLA-4 or CHO-K1 for 30 min. SA-PE 0.5 μL was added for a 30-min incubation after washing with PBS. Representative graph **A** and statistics **B** of affinity between CTLA-4 binding peptides and CHO-K1/hCTLA-4, CHO-K1.GA peptide was the negative control, while anti-hCTLA-4 was the positive control. Fluorescence intensity detected by flow cytometry was used to calculate the binding rate. Representative graph **C** and statistics **D** of blocking experiment. (*p < 0.05, **p < 0.01, ***p < 0.001)

We constructed a CHO-K1 / hCTLA-4 cell line with high CTLA-4 expression to determine whether the affinity peptide specifically binds to CTLA-4 molecules on the cell membrane. CTLA-4 affinity peptides with biotin label were synthesised and co-incubated with CHO-K1/hCTLA-4 cell line. LC4, LC7 and LC8 had affinity with CHO-K1/hCTLA-4 cell line compared with the GA peptide group, which proved that these three peptides could specifically bind with hCTLA-4 at the cellular membrane (Fig. 2).

### Affinity peptides impaired the CTLA-4 and CD80 interaction

**Fig. 3:**
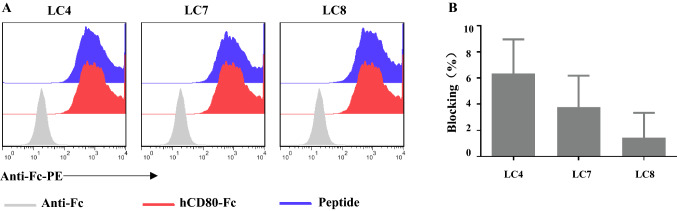
The CTLA-4 binding peptide effect on CTLA-4/CD80 interaction was determined via cell-based blocking assay. CTLA-4 binding peptide at 200 μM was incubated with CHO-K1/hCTLA-4 cells for 30 min, followed by incubation with 10 ng human CD_80_-Fc for 30 min, and finally, anti-human IgG Fc-PE antibody 0.3 μL was added. The fluorescence intensity detected by flow cytometry was used to calculate the blocking rate

Targeting CTLA-4 to treat tumours requires blocking the interaction between CTLA-4 and its CD80/CD86 ligand. The affinity between CTLA-4 and CD80 is higher than that between CTLA-4 and CD86 [13]), and only one of the four amino acids at the binding site is different. Therefore, the next step is to identify whether LC4, LC7 and LC8 could block the CTLA-4 and CD80 interaction. Cell-based blocking assays showed that LC4, LC7 and LC8 could all block the interaction between CTLA-4 and CD80 (Fig. 3). LC8 was abandoned by its weakest blocking effect, and only LC4 and LC7 were retained.

## LC4 plays an anti-tumour role by refreshing local CD8 + T cells and CD4 + T cells, and activates peripheral immune response at the same time

### LC4 activated CD8^+^ T cells and CD4^+^T cells in vitro

**Fig. 4:**
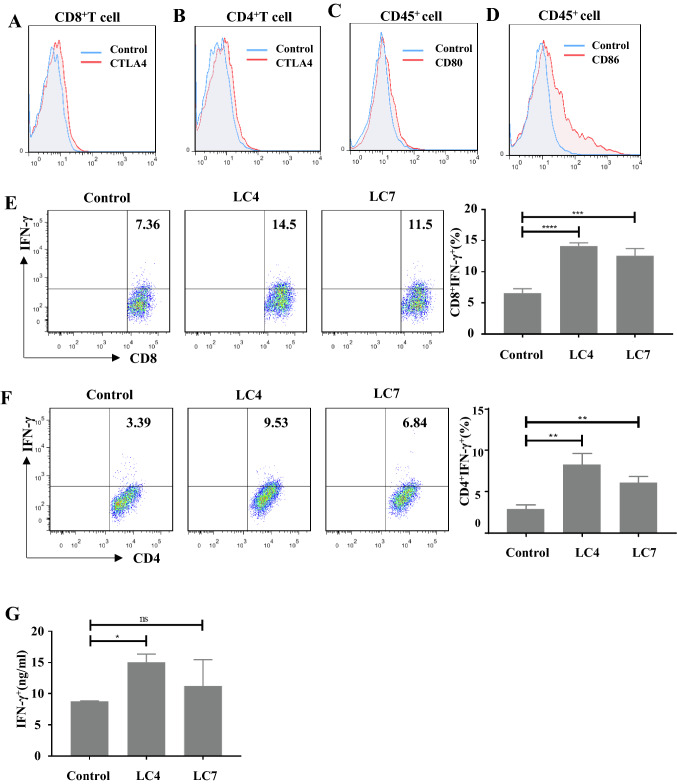
CTLA-4 binding peptides activation on human T cells was determined in vitro. PBMC was added to a 48-well plate at 2 × 10.^5^ cells/well. Anti-Human CD_3_ (1 μg/ml) and CD_28_ (0.5 μg/ml) antibodies were added to stimulate T cell activation, then 200 μM CTLA-4 binding peptide was added to the system and incubated in a 37℃ 5% CO_2_ for 3 days. The expression of CTLA-4 **A**-**B**, CD_80_ **C** and CD_86_ **D** in T cell activation experiment. Effects of CTLA-4 binding peptides on the percentage of **E** CD_8_ + IFN-γ + T cells and **F** CD_4_ + IFN-γ + T cells. **G** Elisa detects the amount of IFN-γ in the cell culture supernatant. (ns = no significance, *p < 0.05, **p < 0.01, ***p < 0.001, ****p < 0.0001)

CTLA-4 could be inducible and expressed on peripheral CD8^+^ T cells and CD4^+^T cells upon activation (Fig. 4A–D). Peripheral blood mononuclear cells (PBMCs) from healthy donor blood were isolated to verify whether LC4 and LC7 can activate T cells in vitro, CD3 and CD28 antibody were added to stimulate T cells and incubated with PBS LC4 or LC7. The IFN-γ secretion amount by the cells was measured using ELISA. IFN-γ production in PBMC cells was significantly enhanced when treated with LC4 (Fig. 4E–G). At the same time, we detected the CD8^+^ T cell and CD4^+^ T cell percentage producing IFN-γ in each T cell subset by flow cytometry. As shown in Fig. 4, LC4 significantly increased both the percentage of CD8^+^ IFN-γ^+^ and that of CD4^+^ IFN-γ^+^ compared with the control. LC7 also had a similar effect, but LC4 was better in activating the T cell ability to secrete cytokines generally.

### LC4 could inhibit CT26 tumour growth but activate peripheral immune response

We compared the Ig V region amino acid sequences of human CTLA-4 protein and mouse CTLA-4 protein to explore the interaction between human CTLA-4 binding peptide LC4 and mouse CTLA-4, and found that the consistency and similarity between them reached 66% and 79%. It has been reported at the same time that there are 11 binding sites between human CTLA-4 and its ligand CD86, while the mouse CTLA-4 binding site only changes at position 105, Tyr changes to Phe [14]. This indicated that human and mouse CTLA-4 protein had high consistency and similarity. LC4 with biotin label were co-incubated with CHO-K1/mCTLA-4 cell line, the results showed that LC4 also has affinity with CHO-K1/mCTLA-4 cell line, which may be the reason why the CTLA-4 site and its ligand interaction is highly conserved. LC4 also increases the CD8 + IFN-γ + T cells and CD4 + IFN-γ + T cells percentage in mouse spleen significantly, and increased the IFN-γ secretion in the supernatant of culture (data were shown in supplementary information). Therefore, it is feasible to verify the anti-tumour LC4 effect in vivo by tumour-bearing model in mice.

**Fig. 5:**
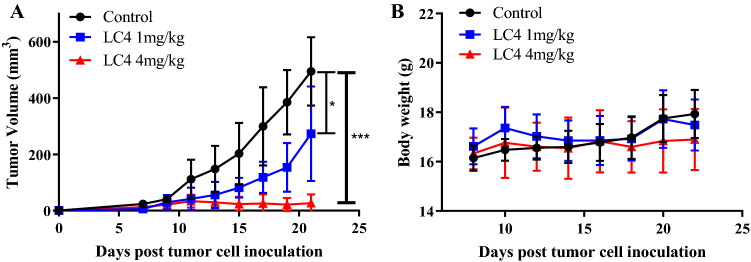
LC4 could significantly inhibit CT26 tumour growth in vivo*.* BALB/c mice were subcutaneously injected with 2 × 10^5^ syngeneic CT26 cells to establish colorectal cancer xenograft model. The mice were divided into control group, LC4 (1 mg / kg) group and LC4 (4 mg / kg) group when the tumor grew to 40–80 mm.^3^, peritumoral administration for 14 days. Statistical graph of **A** tumor volumes and **B** body weight of LC4 (1 mg / kg) group, LC4 (4 mg / kg) group and control group. Data are expressed as the mean ± SD (n = 5) and statistical significance between groups was determined by Student’s t test: *p < 0.05, ***p < 0.001

CT26 tumour-bearing mice were treated with LC4 (1 mg/kg/day or 4 mg/kg/day) for 14 days to identify the in vivo LC4 effect. The results showed that LC4 could significantly inhibit CT26 tumour growth in a dose-dependent manner, but had no significant effect on the weight of mice (Fig. 5A, B).

**Fig. 6:**
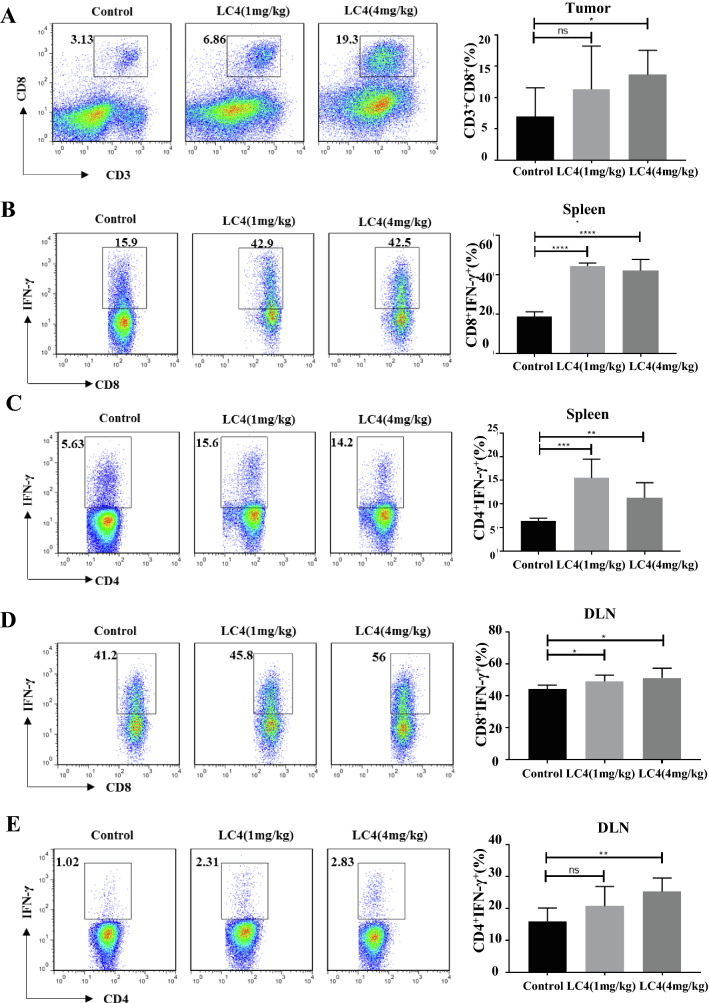
The LC4 activation on T cells was determined in vivo. Tumour tissues were cut into pieces and digested with collagenase IV and DNase I, and then ground into single cell suspension. PMA and ionomycin stimulated the spleen cells, draining lymph node cells of the tumour-bearing mice. Flow cytometry was used to detect the LC4 effect on T cells in vivo. The percentage of **A** infiltrating CD_8_^+^T cells in the tumour, **B** CD_4_^+^IFN-γ^+^T cells in the spleen, **C** CD_8_^+^IFN-γ^+^T cells in the draining lymph nodes, and **D** CD_4_^+^IFN-γ^+^T in the draining lymph nodes. Data are expressed as the mean ± SD (n = 5) and statistical significance between groups was determined by Student’s t test: *p < 0.05, **p < 0.01, ***p < 0.001, ****p < 0.0001; ns = no significance

CD8^+^ T cell and CD4^+^ T cell infiltration were detected in tumours. As a result, CD8^+^ T cell and CD4^+^T cell infiltration in the LC4 group (4 mg/kg/day) significantly increased compared with the control group (Fig. 6A). The percentages of IFN-γ producing CD8^+^ T cells and CD4^+^T cells in the spleen, and draining lymph node were determined. The results showed that both high-dose and low-dose LC4 groups could significantly increase the CD8^+^IFN-γ^+^T cells and CD4^+^IFN-γ^+^T cells ratio compared with the control group (Fig. 6B–E). All these results indicated that although peritumoral administration was selected, LC4 could still activate peripheral immune organs through tissue infiltration and diffusion. Therefore, we preliminarily modified LC4 peptide to improve the LC4 targeting to improve its anti-tumour activity and reduce the impact on peripheral immune organs.

## LC4-PLG-RGD showed stronger antitumor function and lower peripheral immune activation

### LC4-PLG-RGD inhibit CT26 tumour growth more efficiently by refreshing CD8 + T cells and CD4 + T cells in tumour

The tumour targeting of LC4 can be improved by connecting the tumour-targeting peptide RGD, but the simple RGD peptide connection cannot guarantee the LC4 peptide release in the tumour microenvironment. Therefore, LC4-PLG-RGD and RGD-PLG-LC4 were obtained by linking RGD and LC4 with PLGLAG sequence. RGD could bring LC4 into tumour tissue, after PLGLAG digestion by matrix metalloproteinases. LC4 is exposed to realise the targeted delivery. N-terminal RGD-modified peptide RGD-PLG-LC4 and C-terminal RGD-modified peptide LC4-PLG-RGD were synthesised (the amino acid sequences were shown in Table 1).

**Fig. 7:**
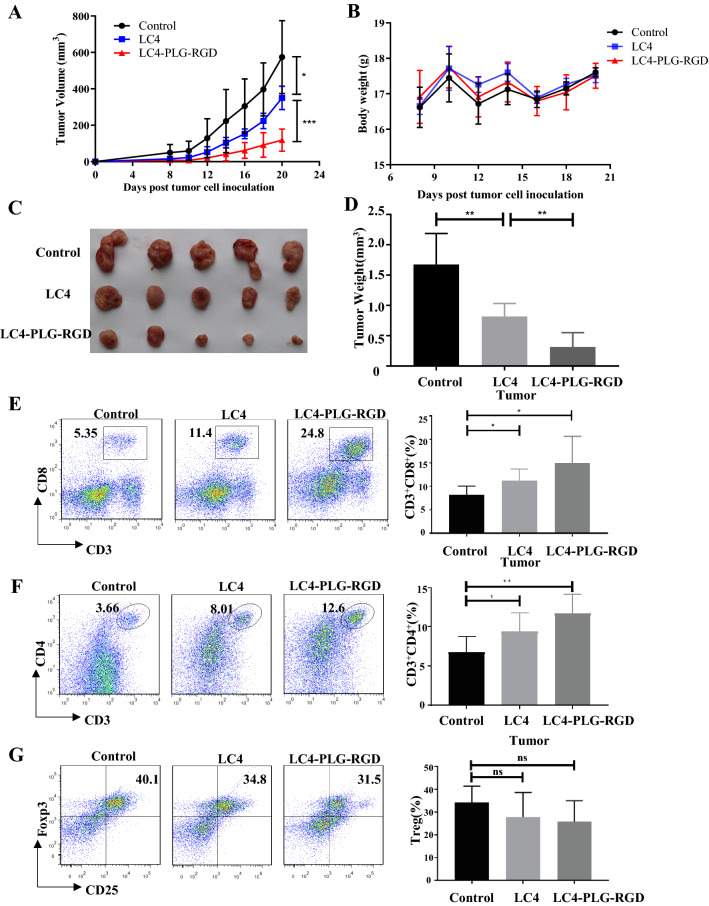
LC4-PLG-RGD inhibit CT26 tumour growth by improving targeting ability. CT26 tumour-bearing mice model was established, tail vein administration, for 14 days. **A**–**D** Statistics of The **A** tumour volumes, **B** body weight, **C** tumour sizes, and **D** tumour weight of LC4-PLG-RGD, LC4 and control group. **E**–**G** The percentage of **E** infiltrating CD8^+^ T cells, **F** infiltrating CD4^+^ T cells and **G** infiltrating Treg cells in the tumour. Data are expressed as the mean ± SD (n = 5) and statistical significance between groups was determined by Student’s t test: *p < 0.05, **p < 0.01, ***p < 0.001

CT26 tumour-bearing mice were treated with LC4 (4 mg/kg/day), LC4-PLG-RGD or RGD-PLG-LC4 (6.2 mg/kg/day) by injected in tail vein for 14 days (Fig. 7A–D). The results showed that LC4, LC4-PLG-RGD and RGD-PLG-LC4 could inhibit CT26 tumour growth. LC4-PLG-RGD had the best anti-tumour effect, followed by RGD-PLG-LC4 (data of RGD-PLG-LC4 have not shown). This shows that LC4 still retains its anti-tumour activity after coupling LC4 with RGD by PLGLAG, and the anti-tumour C-terminal activity coupled with LC4-PLG-RGD is better. The MST binding curves also showed that LC4-PLG-RGD improved the affinity between LC4 and hCTLA-4 better (data were shown in supporting information). Therefore, RGD-PLG-LC4 was abandoned, and only LC4-PLG-RGD was retained for subsequent experiments.

The infiltration of CD8^+^T cells, CD4^+^T cells, and FOXP3^+^Tregs were detected in tumours. As a result, the infiltration of CD8^+^T cells and CD4^+^T cells significantly increased both in the LC4 and LC4-PLG-RGD groups, while the CD4^+^T cells percentage in the LC4-PLG-RGD group was significantly higher than that in the LC4 group (Fig. 7E and F). Meanwhile, there was a tendency to reduce the infiltrating Treg cells in the tumour. This might be because RGD sequence improves peptide targeting. LC4-PLG-RGD targets tumour tissues and has higher concentration in tumour than LC4, so the anti-tumour effect of LC4-PLG-RGD is better than that of LC4 in vivo although LC4-PLG-RGD and LC4 had the same molar concentration.

### LC4-PLG-RGD exhibited lower peripheral immune activation activity

**Fig. 8:**
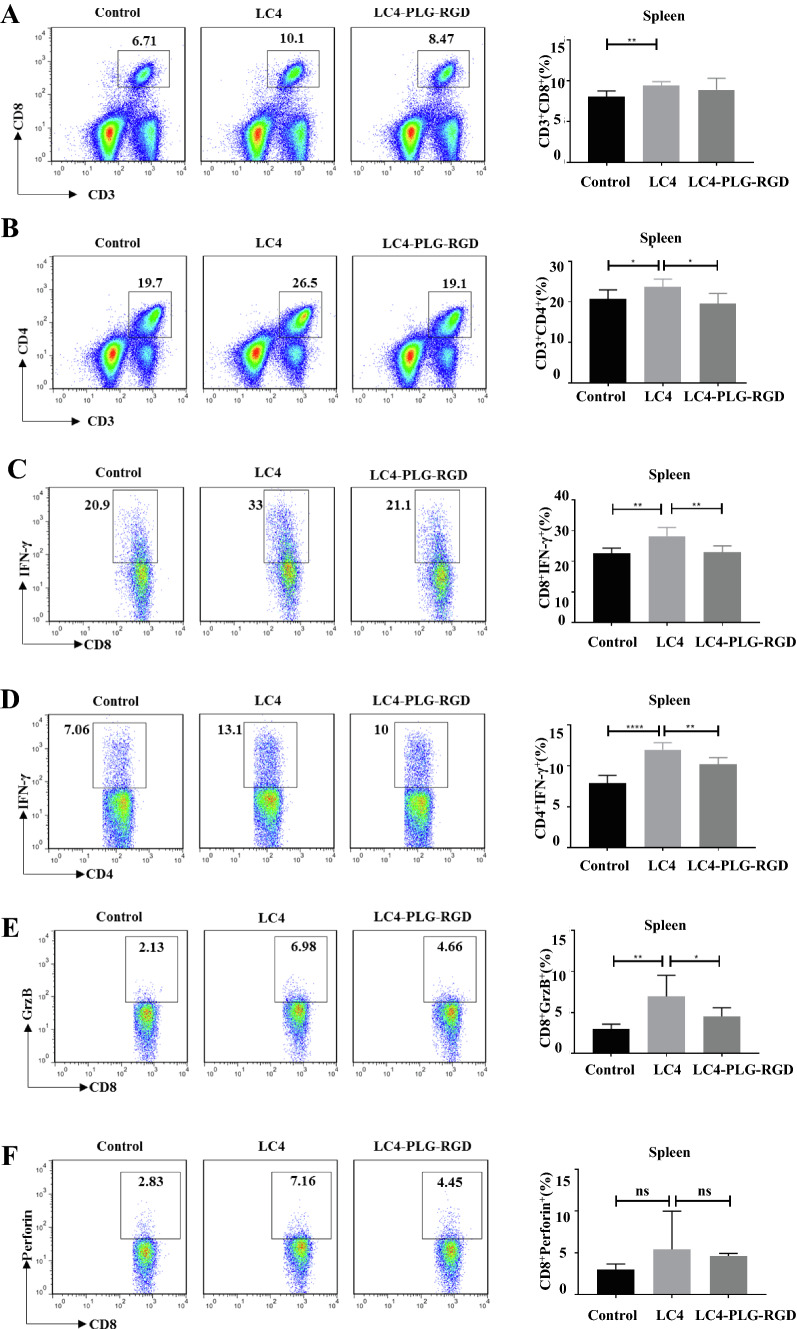
LC4-PLG-RGD stimulate T cells proliferation and cytokine secretion in peripheral immune organs. CT26 tumour-bearing mice model was established, tail vein administration for 14 days and sacrificed 20 days after tumor cell inoculation. **A**–**F** The percentage of **A** CD8^+^T cells, **B** CD4^+^T cells, **C** CD8^+^IFN-γ^+^T cells, **D** CD4^+^IFN-γ^+^T cells, **E** CD8^+^ GrzB^+^ T cells, and **F** CD8^+^Perforin.^+^T cells in the spleen. Data are expressed as the mean ± SD (n = 5) and statistical significance between groups was determined by Student’s t test: *p < 0.05, **p < 0.01, ***p < 0.001, ****p < 0.0001

CD8^+^T cells can kill tumour cells by secreting granzyme B (grzB), perforin, and IFN-γ. CD4^+^T cells can produce direct anti-tumour effects by secreting IFN-γ, We next detected the T cell ability in the spleen and draining lymph nodes to proliferate and secrete cytokines by flow cytometry to figure out whether LC4-PLG-RGD activates peripheral immunity (Fig. 8A–F). The results showed that CD8^+^GrzB^+^T cells, CD8^+^Perforin^+^T cells, CD8^+^IFN-γ^+^T cells, and CD4^+^IFN-γ^+^T cell percentage in the spleen increased in the LC4 group, while the ability to stimulate splenic T cells to secrete cytokines was weaker in LC4-PLG-RGD group than that of the LC4 group. A similar phenomenon was observed when draining lymph nodes (data not shown).

The RGD modified LC4-PLG-RGD ability to stimulate proliferation and T cell secreting cytokines in the peripheral immune organs was weakened compared with LC4, which proved that RGD modification successfully reduced the systemic effect LC4 administration on peripheral immune organs and achieved specific targeting on tumour tissues.

## Discussion

While ICI therapy has improved melanoma patient outcomes, it has also resulted in unique immune-related adverse events (irAEs) rise [15, 16]. The CTLA-4 antibody drug ipilimumab, which has been listed, can produce strong anti-tumour effects in vivo, but antibody drugs are expensive and have strong immunogenic effect, while CTLA-4 activation antibody to the systemic immune system will cause irAEs in a variety of peripheral tissues [17, 18]. IrAEs is the main obstacle limiting ICIs therapy application. Enhancing the anti-tumour checkpoint inhibitor effects and reducing irAE is an urgent problem that needs a solution.

Peptide drugs has the advantage to conveniently synthesise compared with antibody drugs, lower immunogenic response, and transform easily. In this study, the peptide LC4 with specific affinity to the human CTLA-4 protein by phage display was identified. LC4 peptide could effectively block the CTLA-4/B7 protein interactions, activate peripheral immune organ activity while activating T cell activity in tumour tissues. Both LC4 peptide and ipilimumab can effectively block CTLA-4/B7 binding to activate the immune response compared with ipilimumab alone. LC4 could increase CD8^+^T cell percentage in tumour tissue and activate the infiltrating T cell function in tumour tissue in vivo, which shows that LC4 peptide has ICIs activity. The spleen and draining lymph nodes are important peripheral immune organs. On the one hand, their activation is conducive to body anti-tumour, while on the other hand, systemic immunity generation may cause immune-related adverse reactions [19, 20]. Both LC4 high-dose and low-dose groups could significantly increase CD8^+^IFN-γ^+^T cell and CD4^+^IFN-γ^+^T cell percentage in the spleen and drain lymph nodes, which is consistent with the reported ICIs (antibody) effect [19]. Pan Zheng and Yang Liu in 2018 have shown that the main molecular mechanism of CTLA-4 antibody drugs is to clear the local tumour Treg through the ADCC effect mediated by the Fc CTLA-4 segment, rather than by blocking CTLA-4/B7 interaction [21, 22]. However, our results do not support this view. LC4 is a peptide drug without Fc segment, which will not cause ADCC effect in vivo, but LC4 can cause CD8^+^T cell chemotaxis, which means its mechanism needs to be further studied.

CT26 colon tumour model was used to verify the anti-tumour activity of LC4 in vivo, which has the highest immunogenicity, and is the model most responsive to CTLA-4 inhibitor treatment. We took out the CT26 tumour-bearing mouse model’s heart, liver, lung, and kidney, and observed the tissue sections under microscope after H&E staining. No obvious pathological changes were found in the organs of mice after treatment with LC4 (data not shown). Cha E et al. has shown that patients who produce irAEs after treatment with CTLA-4 antibody are characterised by the proliferation of autoreactive CD4^+^ T cells [23]. Dardalhon V et al. has shown that CD4^+^T cells (Th1) secreting IFN-γ are related to the autoimmune response occurrence [24]. In our study, the CD8^+^T cells and CD4^+^T cells secreting IFN-γ ratio in the spleen and draining lymph nodes in CT26 xenograft mouse models significantly increased after treatment with LC4 peptide. This suggested that although no obvious pathological changes were observed by H&E staining, the CD4^+^T cell secreting IFN-γ increase proved that CTLA-4/B7 interaction blocking by LC4 still resulted in irAE increase. At the same time, CD8^+^T cells secreting IFN-γ increase suggested that the peripheral immune organs were indeed activated, which may be accomplished with the assistance of activated CD4^+^T cells. Based on the above results, we next improved LC4 targeting to enable LC4 to carry out local immunotherapy at the tumour tissue in order to reduce peripheral immune system activation.

We coupled RGD and LC4 by use PLGLAG, RGD can bring LC4 into tumour tissue and cut the linker PLGLAG to expose LC4 peptide under the action of matrix metalloproteinases, which has a high expression in tumour cells. Our studies demonstrated that the LC4-PLG-RGD anti-tumour activity is better than LC4 in vivo, which can infiltrate more CD8^+^ T cells and CD4^+^ T cells in tissues, reduce the Treg cell proportion infiltrated by tumour, and weaken the activation ability of T cells in peripheral immune organs. The limitations of this study was only observed the functional change of T lymphocytes in different organizations and the stimulus, according to the data to speculate the immune toxicity reaction, but not to look at the actual toxic effects such as colitis, to directly prove the increase tumor targeting can improve the antitumor activity of the immune checkpoint inhibitors and reduce its peripheral toxicity., and this part needs to be further improved..

In conclusion, a novel peptide LC4 targeting CTLA-4 was identified by phage display bio-panning, which has specific affinity with CTLA-4 and blocks the interaction between CTLA-4 and its CD80 ligand, which inhibits tumour growth. We modified LC4 to obtain LC4-PLG-RGD peptide since it can activate T cells in tumour tissues, as well as T cells in peripheral immune organs, whose ability to stimulate the T cell activation in peripheral immune organs was weakened, and could target tumour tissue while inhibiting tumour growth. Its anti-tumour activity is better than that of LC4. The results of this study suggested that enhancing checkpoint inhibitor tumour targeting can not only enhance their anti-tumour effect, but also effectively reduce their side effects, which is a new strategy in cancer immunotherapy.

## Methods

### Mice

Female 6-week-old BALB/c mice were purchased from Beijing Vital River Laboratory Animal Technology Co., Ltd. (Beijing, China). The animals had free access to food and water and were maintained in a specific pathogen-free facility (24 °C ± 1 °C). Animal welfare and experimental procedures were carried out and approved in accordance with the Ethical Regulations on the Care and Use of Laboratory Animals of Zhengzhou University (Zhengzhou, China).

### Tumour model and treatments

Female BALB/c mice were subcutaneously injected with 1 × 10^5^ syngeneic CT26 cells to establish colorectal cancer xenograft model. Tumour sizes were measured using a digital caliper, and tumour volumes were calculated as Eq. (1):

1 $${\text{V }} = { 1}/{2 } \times {\text{ a }}\left( {{\text{length}}} \right) \, \times {\text{ b }}\left( {{\text{width}}} \right) \, \times {\text{ c }}\left( {{\text{height}}} \right)$$

Treatment of mice was initiated after the tumours had been grown for 8–10 days until reaching a palpable size of 40–80 mm^3^. Tumour-bearing mice were randomly grouped, paraneoplastic LC4 injection and LC4-PLG-RGD was administered via tail vein for 14 days. Normal saline was used as a negative control. The tumour volume was measured every 2 days, and the body mass was weighed.

### Bio-panning of hCTLA-4 protein binding phages

The Ph.D.™ -12 Phage Display Peptide Library Kit was purchased from New England Biolabs (Beijing, China). The panning experiment was conducted according to the manufacturer’s instructions. HCTLA-4 protein 100 μg/ml was coated onto a 96-well plate overnight at 4 °C with gentle agitation in a humidified container in 0.1 M NaHCO_3_ pH8.6 solution. After washing with Tris-buffered saline (containing 0.1% Tween 20), 0.1 M NaHCO_3_ pH8.6 blocking buffer containing 5 mg/mL BSA (Sigmae-Aldrich, Shanghai, China) was added for 3 h incubation at 4 °C. After washing with TBST, 10^13^ pfu phages were added for 1 h incubation at 25 °C with gentle shaking. Then, the unbound phages were removed. Gly-HCl 200 μL 0.2 M (pH2.2 with 0.1% BSA) was used to harvest the binding phages. Bio-panning 84 phage clones were selected randomly and sequenced after five rounds.

### Peptide synthesis

Peptides were synthesised in our lab by using standard solid phase Fmoc synthesis strategy and purified to more than 95% purity by RP-HPLC. Their molecular weights were assessed by mass spectrometry. Peptides were added with *GGGK-biotin* at carboxyl terminal for flow cytometry (FCM) binding assay. Peptide sequences were as follows: GAGAAGGAGGGG*GGGK-biotin* (GA-biotin, control peptide); SWHWHTHVRHQM*GGGK-biotin* (LC3-biotin); WGHSHFSHWKGR*G- GGK-biotin *(LC4-biotin); LDRPSSLAHLAS*GGGK–biotin* (LC7-biotin); RHHSSNP-RDTAP*GGGK-biotin* (LC8-biotin).

### Cell lines and cell culture

Murine colorectal cancer cell lines CT26 were cultured in DMEM medium (GIBCO, Grand Island, NY, USA). DMEM and RPMI 1640 medium were supplemented with 10% FBS (Biological Industries, Kibbutz Beit HaEmek, Israel), 100 μ/mL penicillin (Solarbio, Beijing, China) and 100 mg/mL streptomycin (Solarbio) at 37 °C with 5% CO_2_ under fully humidified conditions.

### Affinity measurements by MST

MST measurements were performed using the Monolith NT.115 system (NanoTemper Technologies GmhH, München, Bayern, Germany) to assess the CTLA-4 affinity binding peptides to Human CD80-Fc protein. The CTLA-4 binding peptides were first diluted to 400 μmol/L with MST buffer, and twofold serial dilutions were carried out to obtain 16 concentration gradients subsequently. Equal Red-NHS647 dye-labelled protein and peptide volumes were incubated at room temperature for 5 min, the mixture was loaded onto standard capillaries (NanoTemper Technologies GmhH), and immediately placed in an MST instrument for detection. The dissociation constant (Kd) was determined using the NanoTemper analysis software MO. Affinity Analysis v2.2.4.

### Peptide binding assay by flow cytometry

In brief, 5 × 10^5^ CHO-K1 cells transfected with pLVX-Puro/hCTLA-4 and pLVX-Puro/mCTLA-4 were used. Cell suspension was incubated with rat serum to block Fc receptors for all flow cytometry assay, and stained with corresponding antibody or biotin-peptides at 4 °C for 30 min, and then analysed by flow cytometry after washing twice.

### Cell-based blocking assay

CHO-K1 cells expressing hCTLA-4 and mCTLA-4 were used for cell-based blocking assay. Briefly, the assay was carried out in PBS pH 7.2, each 50 µL reaction system contained a final CTLA-4 binding peptides 200 µmol/L concentration and 10 ng of Human CD80-Fc protein. The mixture was added to 3 × 10^5^ CHO-K1 cells with further incubation at 4 °C for 30 min after incubation for 30 min at 4 °C. Subsequently, the anti-human IgG Fc-PE (eBioscience) was added and incubated at 4 °C for 30 min, and the cells were washed and analysed by a FACS Calibur flow cytometry (BD Bioscience). The reaction system without peptide was used as a positive control, and the system in which cells only reacted with the flow anti-Fc-PE antibody served as a negative control.

### PBMC function assay

PBMCs from healthy donors were separated by density gradient centrifugation means (Tianjin Hao Yang Biological Manufacture Co., Ltd., Tianjin, China). PBMCs (8 × 10^5^ cells/well) were cultured in 48-well flat-bottomed plates with 500 μL IMDM containing 10% FBS. The PBMCs were stimulated by 1 μg/mLanti-CD3 (eBioscience) and 0.5 μg/mL anti-CD28 antibodies (eBioscience). The activated PBMCs were incubated with 200 μmol/L peptides. Protein transport inhibitor cocktail (0.5 μL, BD Bioscience) was added to each well and incubated over 4 h. Cells were collected and stained with surface markers antibodies anti-human CD4-PerCP-Cy5.5 (OKT4, eBioscience), anti-human CD8-APC (SK1, eBioscience) prior to fixation and permeabilisation. Permeabilized cells were then stained with anti-human anti-Human IFN-γ PE (4S.B3, eBioscience), and analysed by FACS Calibur flow cytometry (BD Bioscience).

Cells were incubated for 5 days for cytokine detection assay. The IFN-γ levels were evaluated by ELISA kit (eBioscience). Briefly, ELISA plate was IFN-γ capture antibody coated with 100 μL/well overnight at 4 °C, aspirated and washed three times with washing buffer (PBS pH 7.2 with 0.05% Tween-20), and then blocked with ELISA/ELISPOT buffer for 1 h at room temperature. The diluted supernatant samples or IFN-γ standard were added into wells and incubated for 2 h at room temperature. Bound biotinylated IFN-γ were detected using avidin-HRP and 1 × TMB solution. Finally, a stop solution of 50 μL/well was added, and the plate optical density was measured at 450 nm using an EMax PlusMicroplate Reader (Molecular Devices, San Jose, CA, USA).

### CD4^+^T cells, CD8^+^Tcells and FOXP3^+^Tregs infiltration in tumour

Tumour-bearing mice were sacrificed on the last treatment day. Tumour tissues were minced and digested in collagenase IV (Invitrogen, Carlsbad, CA, USA) and Dnase I (Sigmae-Aldrich) for 30 min at 37 °C, and passed through a 70-mm cell strainer. Part of the cells were collected and stained with surface markers antibodies after centrifuging, anti-mouse CD45 FITC (30-F11, eBioscience), anti-mouse CD3 PerCP-eFluor 710 (17A2, eBioscience), and anti-mouse CD8 APC (53e6.7, eBioscience) for 30 min at 4 °C to detect CD4^+^T cells and CD8^+^ T cells.

Another cell part was stained with surface markers antibodies anti-mouse CD45 FITC (30-F11, eBioscience), anti-mouse CD4 APC (GK1.5, eBioscience), and anti-mouse CD25 PE (PC61.5, eBioscience) prior to fixation and permeabilisation. Permeabilized cells were then stained with anti-mouse FOXP3PE-Cy7 (FJK-16 s, eBioscience) for 30 min at 4 °C to detect FOXP3^+^ Tregs. The cells were washed and analysed by a FACS Calibur flow cytometry.

### Intracellular cytokine staining assay

Single cell mouse spleen and draining lymph node suspension were prepared by gentle mechanical disruption. Tumour-infiltrating lymphocytes were isolated from tumour tissues. Cells from CT26 xenograft model were stimulated with 20 ng/mL Phorbol 12-myristate 13-acetate (PMA, Sigma eAldrich) and 1 μmol/L ionomycin (Sigma eAldrich) for 4 h. Cells were then stained with surface markers antibodies anti-mouse CD3 PerCP-eFluor710 (17A2, eBioscience), anti-mouse CD8 APC (53e6.7, eBioscience), or anti-mouse CD4 APC (GK1.5, eBioscience) prior to fixation and permeabilisation. Permeabilized cells were then stained with anti-mouse IFN-γ PE (XMG1.2, eBioscience), analysed by FACS Calibur flow cytometry.

### Statistical analysis

Data are shown as mean ± SD and statistical significance between groups was analyzed using Excel according to the 1-tailed Student's test between different groups. *p < 0.05, **p < 0.01, and ***p < 0.001 were considered statistically significant differences.

## Supplementary Information

Additional file1 (PPTX 946 KB)
