# Supplementary material for: A Novel CTLA-4 affinity peptide for cancer immunotherapy by increasing the integrin αvβ3 targeting
Source: Discov Oncol. 2022 Oct 4;13:99. doi: 10.1007/s12672-022-00562-6 (PMC9532478; doi:10.1007/s12672-022-00562-6)
Supplement: Supplementary file 1 — Additional file1 (PPTX 946 KB) [file 12672_2022_562_MOESM1_ESM.pptx]

## Slide 1
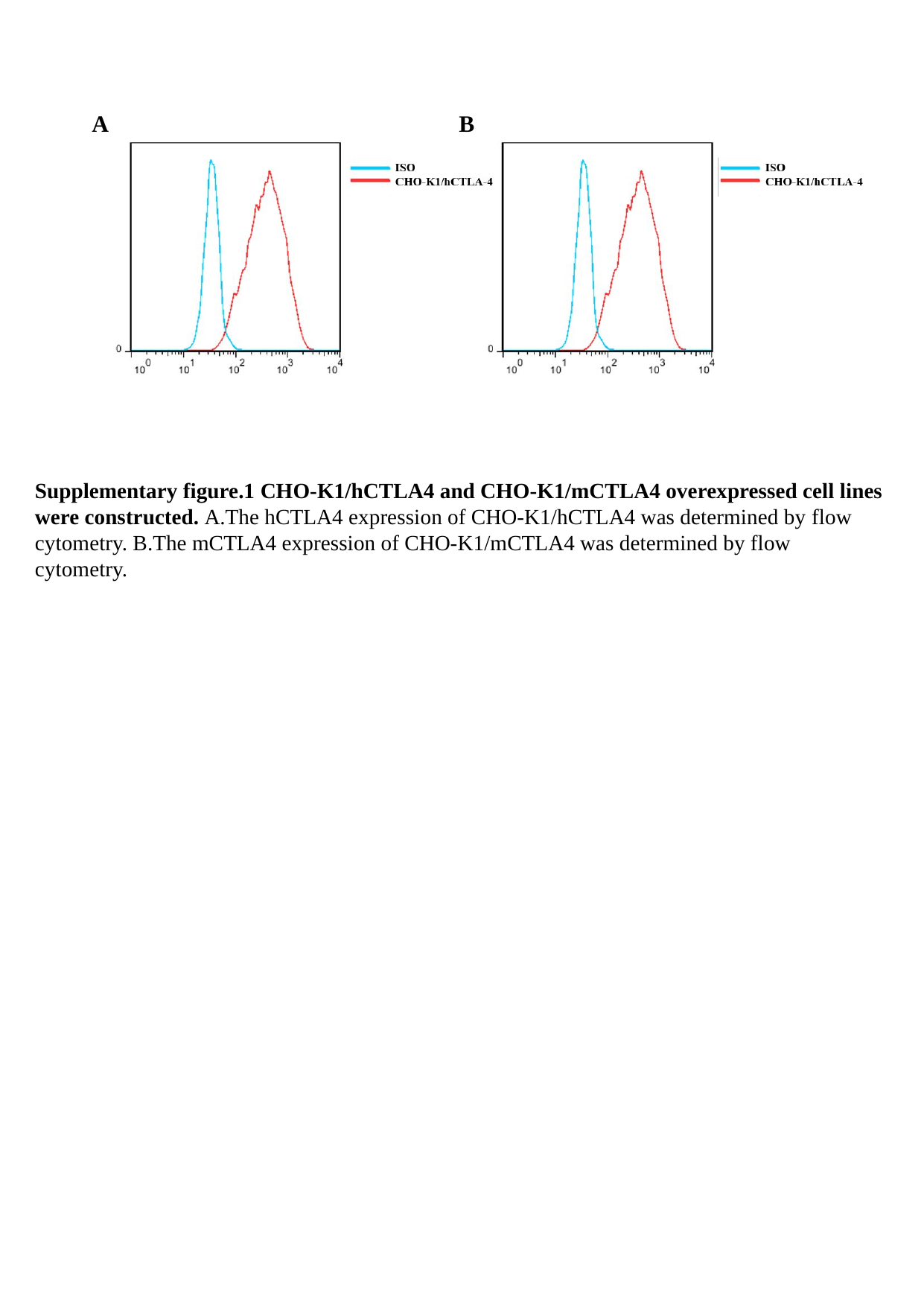

A
B
Supplementary figure.1 CHO-K1/hCTLA4 and CHO-K1/mCTLA4 overexpressed cell lines were constructed. A.The hCTLA4 expression of CHO-K1/hCTLA4 was determined by flow cytometry. B.The mCTLA4 expression of CHO-K1/mCTLA4 was determined by flow cytometry.

## Slide 2
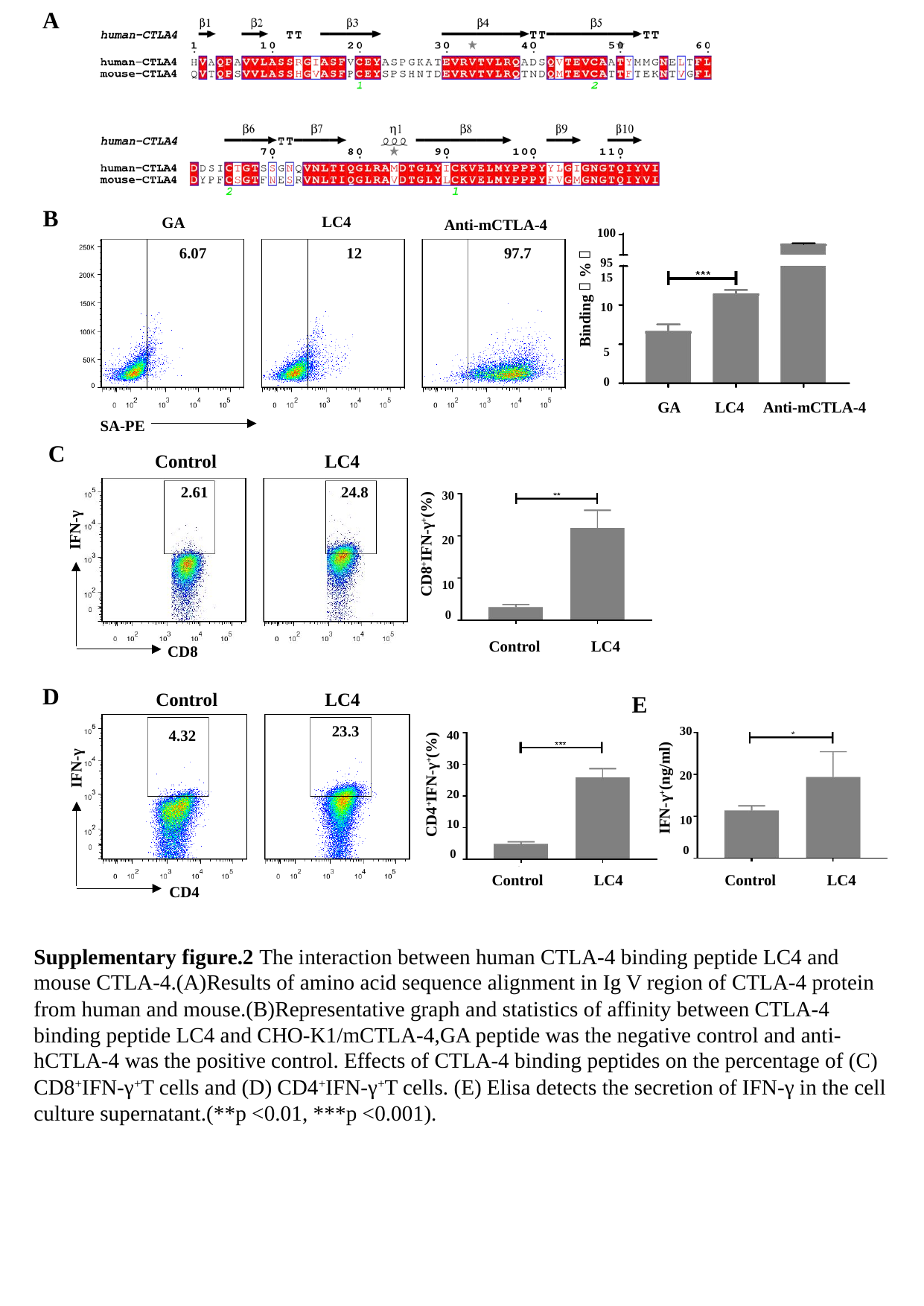

A
B
C
D
Binding（%）
100
95
15
10
5
0
CD8+IFN-γ+(%)
 Control LC4
LC4
GA
Anti-mCTLA-4
6.07
12
97.7
SA-PE
 GA LC4 Anti-mCTLA-4
Control
LC4
IFN-γ
CD8
2.61
24.8
Control
LC4
IFN-γ
CD4
23.3
4.32
E
CD4+IFN-γ+(%)
 Control LC4
 Control LC4
30
20
10
0
IFN-γ+(ng/ml)
40
30
20
10
0
30
20
10
0
Supplementary figure.2 The interaction between human CTLA-4 binding peptide LC4 and mouse CTLA-4.(A)Results of amino acid sequence alignment in Ig V region of CTLA-4 protein from human and mouse.(B)Representative graph and statistics of affinity between CTLA-4 binding peptide LC4 and CHO-K1/mCTLA-4,GA peptide was the negative control and anti-hCTLA-4 was the positive control. Effects of CTLA-4 binding peptides on the percentage of (C) CD8+IFN-γ+T cells and (D) CD4+IFN-γ+T cells. (E) Elisa detects the secretion of IFN-γ in the cell culture supernatant.(**p <0.01, ***p <0.001).

## Slide 3
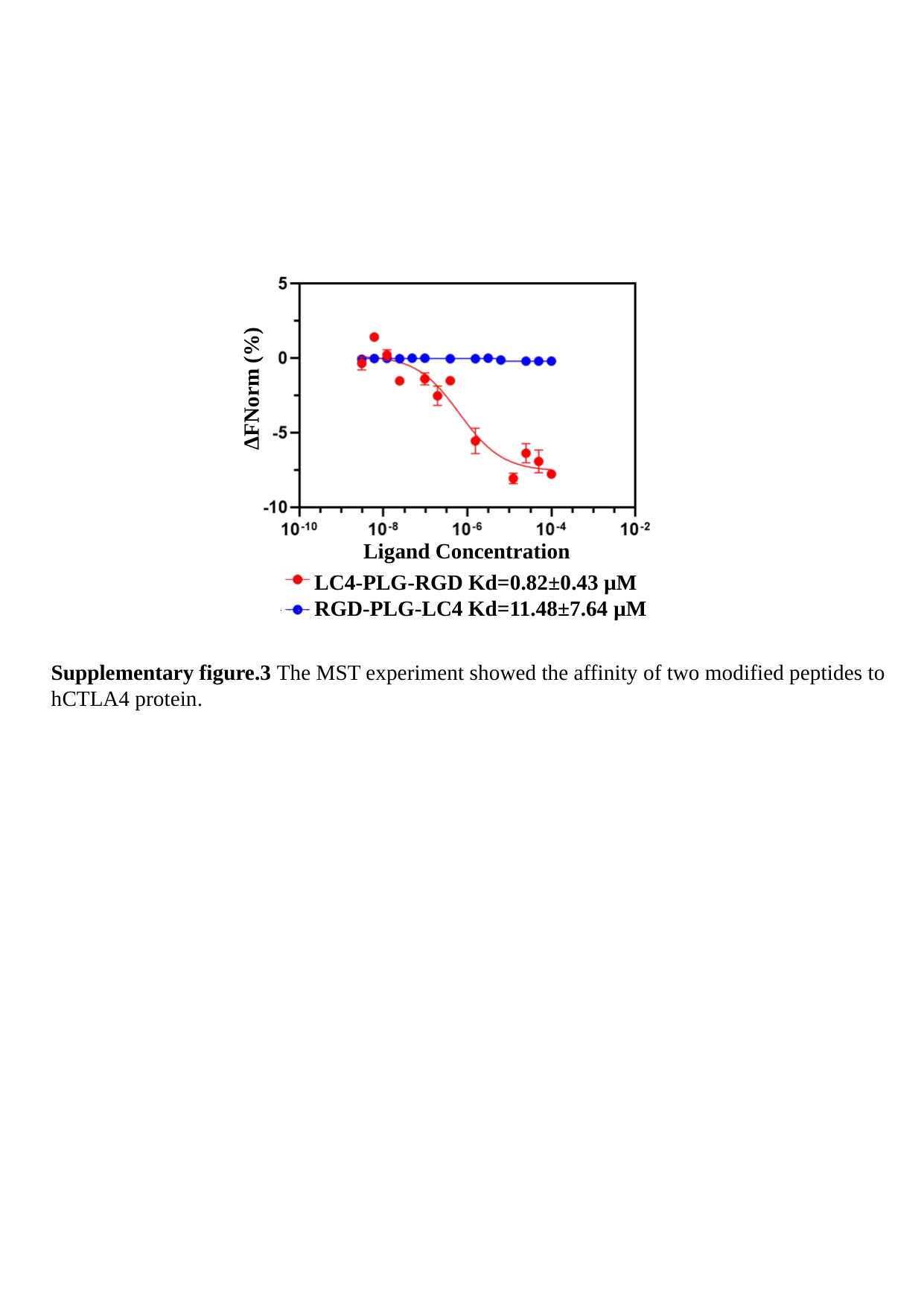

∆FNorm (%)
LC4-PLG-RGD Kd=0.82±0.43 µM
RGD-PLG-LC4 Kd=11.48±7.64 µM
Ligand Concentration
Supplementary figure.3 The MST experiment showed the affinity of two modified peptides to hCTLA4 protein.

## Slide 4
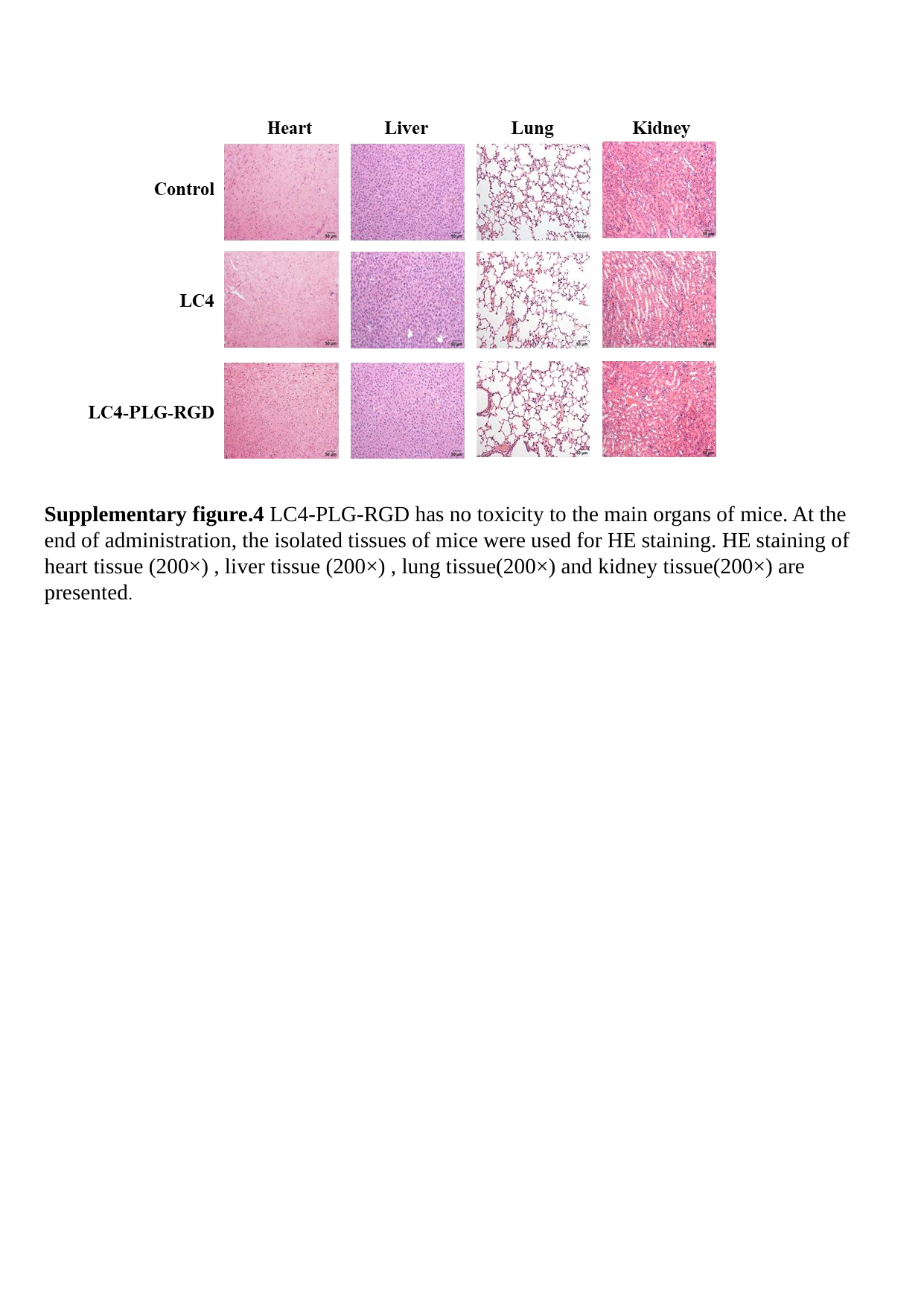

Supplementary figure.4 LC4-PLG-RGD has no toxicity to the main organs of mice. At the end of administration, the isolated tissues of mice were used for HE staining. HE staining of heart tissue (200×) , liver tissue (200×) , lung tissue(200×) and kidney tissue(200×) are presented.
